# Immense Essence of Excellence: Marine Microbial Bioactive Compounds

**DOI:** 10.3390/md8102673

**Published:** 2010-10-15

**Authors:** Ira Bhatnagar, Se-Kwon Kim

**Affiliations:** 1 Department of Chemistry, Pukyong National University, Busan 608-737, Korea; E-Mail: ibhatnagar@gmail.com; 2 Marine Bioprocess Research Center, Pukyong National University, Busan 608-737, Korea

**Keywords:** marine microbes, bacteria, fungi, marine natural products, secondary metabolites, bioactivity

## Abstract

Oceans have borne most of the biological activities on our planet. A number of biologically active compounds with varying degrees of action, such as anti-tumor, anti-cancer, anti-microtubule, anti-proliferative, cytotoxic, photo protective, as well as antibiotic and antifouling properties, have been isolated to date from marine sources. The marine environment also represents a largely unexplored source for isolation of new microbes (bacteria, fungi, actinomycetes, microalgae-cyanobacteria and diatoms) that are potent producers of bioactive secondary metabolites. Extensive research has been done to unveil the bioactive potential of marine microbes (free living and symbiotic) and the results are amazingly diverse and productive. Some of these bioactive secondary metabolites of microbial origin with strong antibacterial and antifungal activities are being intensely used as antibiotics and may be effective against infectious diseases such as HIV, conditions of multiple bacterial infections (penicillin, cephalosporines, streptomycin, and vancomycin) or neuropsychiatric sequelae. Research is also being conducted on the general aspects of biophysical and biochemical properties, chemical structures and biotechnological applications of the bioactive substances derived from marine microorganisms, and their potential use as cosmeceuticals and nutraceuticals. This review is an attempt to consolidate the latest studies and critical research in this field, and to showcase the immense competence of marine microbial flora as bioactive metabolite producers. In addition, the present review addresses some effective and novel approaches of procuring marine microbial compounds utilizing the latest screening strategies of drug discovery.

## 1. Introduction

Microorganisms, including certain bacteria, fungi and algae, produce secondary metabolites which may have some degree of bioactivity, either against another microorganism or acting against certain physiological states of a diseased body. These metabolites, otherwise known as bioactive substances, are profoundly used as antibiotics and may be effective against infectious diseases such as HIV-1 [1]; conditions of multiple bacterial infections (penicillin, cephalosporines, streptomycin, and vancomycin); or neural tube defects and neuropsychiatric sequelae [2,3]. Some drugs have also been found to be useful against carcinomas (bleomycin, dactinomycin, doxorubicin and staurosporin), risk of coronary heart disease, or may act as immune-suppressants (cyclosporin) to aid in organ transplantation [4]; thus making the microbial secondary metabolites an enormous source of pharmaceutical importance. Since the 1920s when the first antibiotic Penicillin was discovered, it was believed that soil microorganisms are the largest source of novel drugs. It is their biological and chemical diversity, coupled with the underlying competence to produce novel secondary metabolites with antimicrobial and nutritive effects, that has led to the widespread use of microorganisms in the economic, industrial scale production of drugs.

The ocean is the mother of life and it is believed that the most primitive forms of life originated from this “primordial soup”. It harbors a vast variety of marine organisms that are diverse in their physiology and adaptations. These marine inhabitants have served as a model for various studies ranging from the deep knowledge about nerve transmission (squid and its giant nerve axons) to the mesenteries of vision (eyes of horseshoe crabs, sharks and skates). The surf clam is being used as an excellent model for the cell cycle and its regulation while the sea urchin is a model for understanding the molecular basis of cellular reproduction and development [5]. As they thrive in a different kind of climate, these organisms develop certain adaptation mechanisms which may be useful for their defense and the result of these adaptations may be useful for human beings in many forms. Production of bioactive metabolites is one such adaptation mechanism, which helps in survival from predators. Some of the bioactive compounds from marine sources will be dealt with in detail later in this review.

It is noteworthy that marine sources have also demonstrated tremendous abilities as producers of anti-cancer compounds and secondary metabolites which act against infectious diseases and inflammation. Blunt *et al.* (2004) listed that in marine environment sponges (37%), coelenterates (21%) and microorganisms (18%), are major sources of biomedical compounds, followed by algae (9%), echinoderms (6%), tunicates (6%), molluscs (2%) bryozoans (1%), *etc.* [6]. However, marine microorganisms have not been given the attention they deserve and a very limited insight into the capabilities and bioactive potential of marine microorganisms is available in literature to date. There is still scope for a higher magnitude of research and investigation to explore the potential of both marine organisms and marine microorganisms as producers of novel drugs. Apart from synthetic products, pharmaceutical industries in most of the developed and developing countries are now concentrating on natural products derived from marine microorganisms (Table 1).

This review focuses on the tremendous potential of marine microbes as prolific producers of bioactive substances and discusses the possible ways to exploit the vast marine microbial treasure for their utilization as novel drug delivery systems.

## 2. Marine Environment as a Prolific Source of Bioactive Compounds

The Primordial Soup Theory suggests that life began in a body of water, possibly a pond or ocean when the chemicals from the atmosphere combined with some form of energy. This combination gave rise to the building blocks of proteins- the amino acids and may have led to the evolution of new species. Hence, oceans may be considered to be rich in organic compounds favorable for the evolution and growth of life in general. It was in the early 1960s that researchers began to concentrate on oceans as a novel and unexplored source of potentially useful bioactive compounds. The basis of this could be the fact that more than 95% of the Earth’s biosphere is ocean [25] and scientists wish to unearth bioactive compounds in unexpected places as the antibiotic resistance increases and the production of novel bioactive compounds tapers down. As a result, more than 10,000 marine metabolites have been isolated and characterized over the past five decades [26].

Rajaganapathi and colleagues (2002) purified and characterized an anti-HIV protein (MW 60 KDa) from the purple fluid of a sea hare and named it “Bursatellanin-P” after the animal species *Bursatella leachii*. This protein was resistant to digestion by proteinase K and mercaptoethanol [27]. Another report on the antiviral activity of a marine microorganism was published in 2006. Wang *et al.* (2006) observed that serum hepatitis B surface antigen was significantly lowered in *Styela plicata* treated mice compared with mice receiving a normal diet. They concluded that *S. plicata* may be an effective antiviral medicine in treating chronic hepatitis B [28].

**Figure 1:**
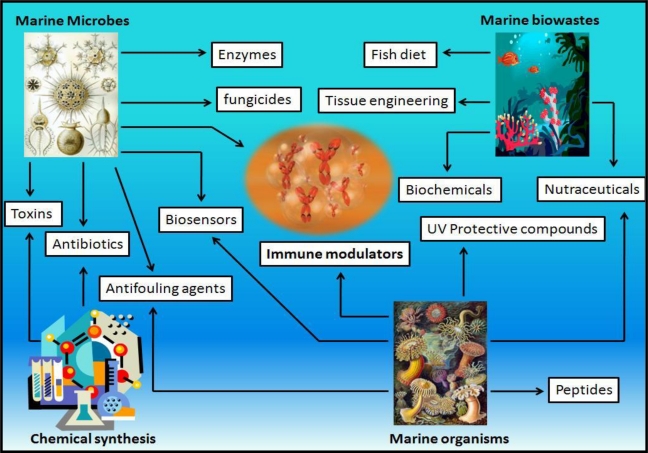
Diversity of bioactive substances produced in the marine environment.

Most of the Earth’s microbial diversity is found in the ocean, which ultimately directs an enormous number of bioactive substances (Figure 1). The various inhabitants of the marine environment including bacteria, fish, algae, corals, crustaceans and even sea mud are known to have tremendous potential to be utilized as cosmeceutical agents [29]. Recently, Ryu *et al.* (2009) demonstrated that methanol extracts of alga *Corallina pilulifera* possessed a high phenolic content, which reduced the expression of UV-induced MMP-2 and -9, in human dermal fibroblast in a dose dependent manner, thereby attaining the capability of inhibiting free radicals [30]. There has been another study on the photoprotective effect of phlorotannins from *Ecklonia cava* against the photo-oxidative stress induced by UV-B irradiation [31].

**Figure:**
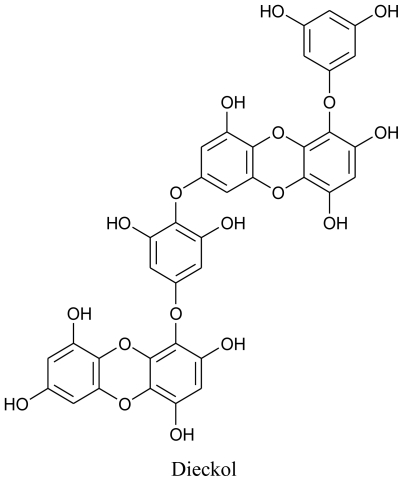


According to their postulations, among the isolated phlorotannins, dieckol showed prominent inhibitory activity against melanogenesis and effectively reduced UV-B radiation-induced cellular damage, which further indicated that dieckol from *E. cava* can be used as an effective natural source to make cosmeceutical or pharmaceutical products. Dieckol

## 3. Marine Microorganisms as Producers of Bioactive Compounds

Microbiology has developed as a great field of science in the recent past with microorganisms being used in almost all arenas. For the past five decades or more, it is the soil micro biota that has been subjected to extensive investigation all over the globe. One good reason for this may be the ease of microbial isolation from lithosphere compared to any other spheres of earth. Owing to the vast variety of novel chemical compounds from these microbes, millions of strains have been employed for pharmacological screening. This is now changing as the rate of development of novel drug agents has slowed down and most of the terrestrially isolated microbial chemical formulations appear repetitive, and are further proving to be costly.

A unique source in the world’s oceans, the deep ocean and the geothermal vents, is now becoming the focus of considerable microbiological interest [32]. Marine micro biota does not only lead in quantitative measures, but also has tremendous potential to represent a biomedical resource of unknown magnitude and great promise. Presence of obligate barophiles, bacteria which require pressures as high as 600 atmospheres for growth has been reported in recent past from the deep sea environment [33]. As certain classes of microbes have unique adaptations for high salt environments and high hydrostatic pressure, the immense miscellany of the microorganisms in marine habitats is quite perceptible. In addition, Inagaki *et al.* (2006) reported that previously unidentified prokaryotic communities such as the JS1 and DSAG groups occur widely in organic rich deep marine sediments associated with methane hydrates along the Pacific Ocean margin. They also suggested that microbial communities can be stratified in deep marine sediments, and surrounding geochemical and geological settings strongly affect the community structure [34]. It is becoming more and more evident that many classes of microorganisms exist only in the sea.

Due to limited literature on the culturing techniques and media formulation for culturing marine microbes, pharmaceutical industries have not been able to fully utilize this enormous resource. The myth still prevails that marine microorganisms are difficult to culture, if not uncultivable. However, a number of reports in recent past have proven that marine microorganisms can now be cultured successfully [25,35–37]. Most of the developed and underdeveloped countries have since shifted their focus to the marine habitat and new marine oriented programs are emerging worldwide. Major classes of microbes like bacteria and fungi are now the target of biomedical study and intriguing novel metabolites are being produced. Coastal bacterial samples grown under saline conditions have been reported to yield new antibiotics, antitumor, and anti-inflammatory compounds [38–40]. In fact, the symbiotic microbial consortia also prove to be a source of bioactive compounds with pharmaceutical potential. Bacteria and fungi have been sampled from the surfaces of marine plants and the internal tissues of invertebrates and, in particular, marine fungi seem to be of increasing interest [41–45].

### 3.1. Marine bacteria

Antagonism is nature’s own counter action for survival and existence. Bacteria produce some secondary metabolites for their defense against other microorganisms and these secondary metabolites serve as a source of bioactive compounds for use in human therapies. Marine bacteria are prolific producers of such secondary metabolites as they thrive in harsh oceanic climates.

*Pseudomonas* are gram-negative Gammaproteobacteria dwelling on lithosphere as well as in a marine environment. Compared to terrestrial isolates, the marine isolates are not well explored and only a limited number have been reported as producers of bioactive compounds. In fact the exploration of marine environment continues for isolation of more novel stains of *Pseudomonas* which are probable sources of bioactive compounds. Recently, Romanenko *et al.* (2008) have isolated and taxonomically classified an aerobic, nonpigmented bacterium strain KMM 3042. It was found to cluster adjacent to *P. borbori* in the *Pseudomonas* genus, with 97% sequence homology. Although few, the chemical structures of these marine *Pseudomonas* derived bioactive substances are diverse, including pyrroles, pseudopeptide pyrrolidinedione, phloroglucinol, phenazine, benzaldehyde, quinoline, quinolone, phenanthren, phthalate, andrimid, moiramides, zafrin and bushrin [46]. Some of these bioactive compounds are antimicrobial agents, and dibutyl phthalate and di-(2-ethylhexyl) phthalate have been reported to be cathepsin B inhibitors [47].

**Figure:**
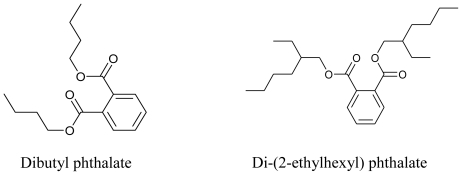


The first report on antimicrobial activity of *Stenotrophomonas* strains isolated from deep sea invertebrates came in 2008, stating that remarkable fungal inhibitory activity was observed in six *Stenotrophomonas* strains isolated from sponge, sea urchin, and ophiura specimens. Although negligible activity was observed against *Candida albicans*, these strains could substantially inhibit Gram-positive microorganisms. It is worth noting here that *S. maltophilia* is an opportunistic pathogen known for its bio-controlling capabilities [48]. Another study from the same group, directed against low molecular weight antimicrobial metabolites from marine ark shell *Anadara broughtoni* associated heterotrophic bacteria, revealed antimicrobial, hemolytic and surface activities in butanol extracts of cells or cell-free supernatant of six active strains [49].

Yet another recently discovered genus of bioactive substance producing marine bacteria is *Pseudoalteromonas.* It was previously misclassified under *Alteromonas* genus and misidentified as *Pseudomonas* or *Chromobacterium* [50]. A couple of years ago, the seawater species *P. phenolica* was reported to inhibit methicillin resistant *Staphylococcus aureus* strains due to a brominated biphenyl compound, 3,3’,5,5’-tetrabromo-2,2’-diphenyldiol [51]. Some strains of *P. luteoviolacea* also seem to have activity against protists [52]. Egan and coworkers (2002) reported that the yellow pigment of *Pseudoalteromonas tunicata* has anti-fungal activity [53] which was later identified as a tambjamine (4-methoxypyrrole-containing bioactive compounds) like alkaloid and designated as YP1 [54] by Franks *et al.* (2005). These tambjamines have been isolated from marine invertebrates and have been previously reported to possess antimicrobial, antitumorigenic, immunosuppressive, anti-proliferative and ichthyodeterrent activities [55]. Evidence points towards the colonizing bacteria at the surface of higher organisms as the source of these compounds [56]. This has further been proven by Burke and colleagues by elucidation of YP1 biosynthetic pathway in *P. tunicata* [57].

**Figure:**
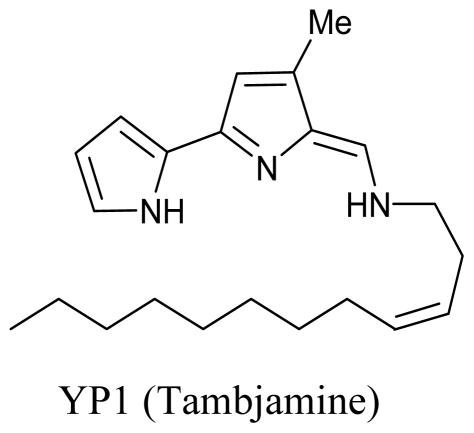


Bacterial polysaccharides are another class of pharmaceutically important secondary metabolites. It has been found that exopolysaccharides (EPS) from marine bacteria contain novel formulations with a variety of properties like thickening, coagulating, adhesion, stabilizing and gelling, which makes them suitable candidates for industrial applications. Moreover, good viscosity and pseudoplastic properties of EPS render them resistant to the extremities of temperature, pH and salinity making them more industry friendly [58]. In a recent study, an EPS-2 having immunomodulatory and antiviral effects on immunocompetent cells, has been reported from *Geobacillus thermodenitrificans* obtained from a shallow marine vent of Volcano Island (Italy) [59].

Bacteria not only produce secondary metabolites against other organisms, but they also produce certain compounds which help in cleaning their environment. Certain marine bacterial species are known as prolific producers of biosurfactants, bioemulsifiers and exopolysaccharides. Das *et al.* (2008) studied the degradation of a model polyaromatic hydrocarbon, anthracene, an organic pollutant, by a marine *Bacillus circulans* strain. In addition, the produced biosurfactant depicted a high degree of emulsification of various hydrocarbons. It was observed that this bacterium utilized anthracene as a carbon substrate for the production of biosurfactant [60]. This group also investigated another biosurfactant producing marine bacterium with an ability to remove metal from solutions with efficiency dependent on the concentration of the metal as well as on the biosurfactant [61].

### 3.2. Marine fungi

Marine derived fungi have been widely studied for their bioactive metabolites and have proven to be a rich and promising source of novel anticancer, antibacterial, antiplasmodial, anti-inflammatory and antiviral agents [62,63]. Some marine fungi have unique new carbon frameworks which are exceptional in nature. Compounds produced by such fungi are of interest as new lead structures for medicine as well as for plant protection. Responding to the great demand for natural compounds from marine derived fungi, Kjer *et al.* (2010) have defined a detailed protocol for their isolation and cultivation from various marine organisms (sponges, algae and mangrove plants) in order to characterize and elucidate the structure of secondary metabolites produced by these fungi [64]. A couple of years ago, Du *et al.* (2007) isolated a novel anthraquinone derivative with naphtho[1,2,3-de]chromene-2,7-dione skeleton, and named it aspergiolide A. It was isolated from a marine filamentous fungus, *Aspergillus glaucus* in the Fujian province of China, and was found to exhibit cytotoxicity against K562 and P388 cell lines [65]. The same group has recently worked on the antitumor activities of alkaloids isolated from a *Penicillium sp.* derived from deep ocean sediment. They isolated two new meleagrin analogs, meleagrin D and E, and two new diketopiperazines, roquefortine H and I which showed weak cytotoxicity as compared to the previously reported meleagrin B and meleagrin that induced HL-60 cell apoptosis or arrested the cell cycle through G2/M phase, respectively. They proposed that the distinct substitutions on the imidazole ring could have a significant influence on the cytotoxicity of these alkaloids [16].

A number of novel compounds and metabolites with bioactive potential continue to be isolated and characterized from marine derived fungi, which are capable of producing not only antimicrobial but also antifouling compounds. In 2006, a bioassay-guided isolation and purification procedure was used to obtain a novel antifouling and antimicrobial compound from a marine-derived fungus *Ampelomyces sp*. The isolate, 3-chloro-2,5-dihydroxybenzyl alcohol effectively inhibited larval settlement of the tubeworm *Hydroides elegans* and of cyprids of the barnacle *Balanus amphitrite* and was non-toxic; suggestive of a potent antifoulant and/or antibiotic activity[66]. Another study from the same group concerned the antibiotic and antifouling compound production by the marine derived fungus *Cladosporium* sp. F14. They reported that in nutrient enriched cultivation media, this strain produced antibiotic and antifouling compounds in the presence of glucose or xylose [67]. In the search for novel antimitotic and antifungal substances from marine-derived fungi, Gai and coworkers (2007) reported that low concentration of the EtOH extracts of the culture broth of a *Fusarium* sp. (strain 05JANF165) were bioactive. Their search for the basis of this bioactivity led to the identification and purification of a new antifungal antibiotic and the chemical structure was elucidated as Fusarielin E [68].

**Figure:**
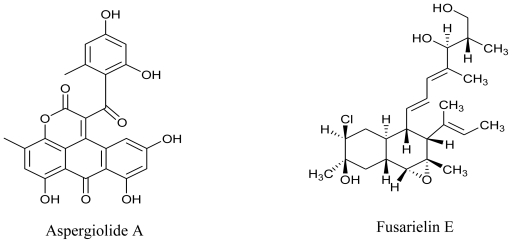


A Korea based study resulted in the isolation of a novel antibacterial dioxopiperazine, dehydroxybisdethiobis-methylthio-gliotoxin, and the previously reported bisdethiobis-methylthio-gliotoxin and gliotoxin, from the broth of a marine derived fungus of the genus *Pseudallescheria* and its structure was assigned through NMR. All three compounds exhibited potent antibacterial activity against the methicillin resistant and multidrug resistant *Staphylococcus aureus*, whereas Gliotoxin showed a significant radical scavenging activity against 1,1-diphenyl-2-picrylhydrazyl (DPPH) with IC_50_ value of 5.2 μM [69].

**Figure:**
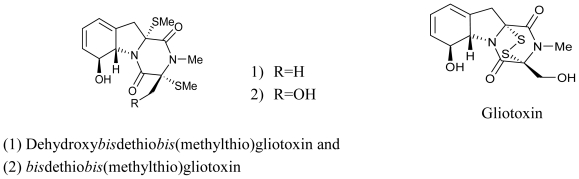


Another study from the same source, reported two novel antibacterial aspyrone derivatives *viz*. Chlorohydroaspyrones A and B, and the previously described aspyrone, asperlactone, and penicillic acid from the broth of a marine isolate of the fungus *Exophiala* and were found to have mild antibacterial activity against *Staphylococcus aureus* [70].

**Figure:**
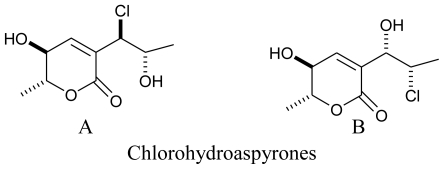


Marine fungi have also been reported to have a nematicidal effect, for example, nematicidal and antimicrobial metabolites were reported previously from marine ascomycete *Lachnum papyraceum* (Karst.) Karst III and the production of novel isocoumarin derivatives was achieved under halogenated Chlorohydroaspyrones conditions [71]. Inspired by this work, Nenkep *et al.* (2010) recently reported the isolation of halogenated benzoquinones (bromochlorogentisylquinones A and B), with significant radical scavenging activity against DPPH, from a marine derived *Phoma herbarum* strain [72].

**Figure:**
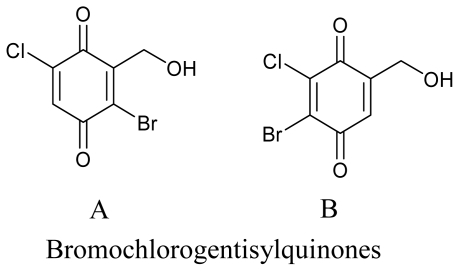


### 3.3. Actinomycetes

Actinomycetes from natural sources are widely recognized to produce secondary metabolites, including many antimicrobials such as streptomycin, erythromycin, and tetracycline, with original and ingenious structures and potent biological activities [73]. Therefore, actinomycetes are considered to be a potent resource for new lead compounds in drug development. Many marine isolates of actinomycetes have been reported to be producers of novel antitumor [74], antimalarial [75] and antimicrobial agents [76,77].

In the exploration of marine derived actinomycetes as a source of antitumor compounds, Cho *et al.* (2007) isolated four new 3-methyl-4-ethylideneproline-containing peptides, Lucentamycins A–D from the fermentation broth of a marine derived actinomycete, *Nocardiopsis lucentensis* (strain CNR-712). Out of the four compounds, Lucentamycins A and B were observed to have significant *in vitro* cytotoxicity against HCT-116 human colon carcinoma [78]. In a report published last year, five isoquinoline quinones, four new derivatives, Mansouramycin A–D, and the known 3-methyl-7- (methylamino)-5,8-isoquinolinedione were isolated from the ethyl acetate extract from the marine derived Mei37 isolate of *Streptomyces* sp. These isolated compounds, when subjected to cytotoxicity analysis against 36 tumor cell lines, indicated significant cytotoxicity with great degree of selectivity for non-small cell lung cancer, breast cancer, melanoma, and prostate cancer cells [79] suggesting their potential as anticancer drugs.

**Figure:**
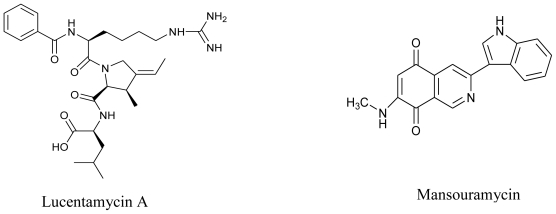


In the same year, Perez and coworkers isolated a macrodiolide Tartrolon D from the fermentation broths of *Streptomyces* sp. MDG-04-17-069. The tartrolons are a class of compounds that have Bromochlorogentisylquinones attracted a great deal of attention owing to their interesting biological properties. The isolated tartrolon in this study was found to display strong cytotoxic activity against three human tumor cell lines *viz*. lung (A549), colon (HT29), and breast (MDA-MB-231) [80]. Yet another study reported the secondary metabolites of a marine *Saccharomonospora* sp. collected at the La Jolla Submarine Canyon. Chemical examination yielded a novel alkaloid Lodopyridone, which was found to be cytotoxic (IC_50_ = 3.6 μM) to HCT-116 human colon cancer cells [81].

**Figure:**
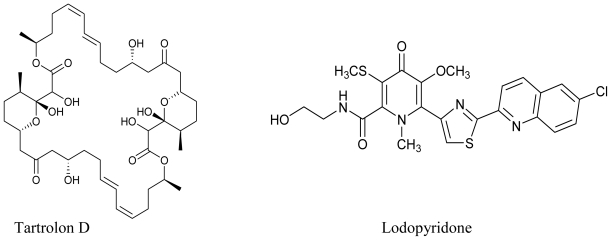


Marine actinomycetes are not only known for their anti-tumor and anti-cancerous potential, they are well documented as antimicrobial agents too. A California based study was successful in isolating a series of chlorinated bisindole pyrroles, Lynamicins A–E from a novel strain of a marine actinomycete, *Marinispora*. This isolate from marine sediment collected off the coast of San Diego (California) demonstrated broad-spectrum activity against both Gram-positive and Gram-negative organisms. When tested for their antimicrobial spectrum against a panel of 11 pathogens, these compounds showed activity against drug-resistant pathogens such as methicillin-resistant *Staphylococcus aureus* and vancomycin-resistant *Enterococcus faecium* [77]. Carlson *et al.* (2009) have reported the isolation of novel dienoyl tetramic acids tirandamycin C and tirandamycin D with activity against vancomycin-resistant *Enterococcus faecalis*, from the marine environmental isolate *Streptomyces* sp. 307-9. These compounds were structurally similar to the previously identified compounds Tirandamycins A and B with a slight variation in the pattern of pendant oxygenation on the bicyclic ketal system [82].

**Figure:**
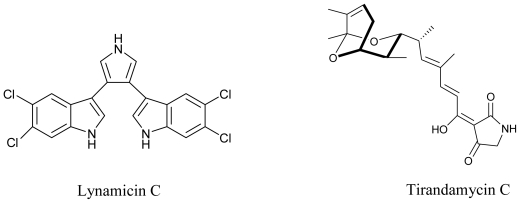


Maskey *et al.* (2004) isolated Trioxacarcins A, B and C along with three new derivatives designated as Trioxacarcins D, E and F, from the ethyl acetate extract of *Streptomyces* sp. isolate B8652. All trioxacarcins showed high anti-bacterial activity whereas some of them showed high anti-tumor and anti-malarial activity [83]. In a latest study, crude extract of a marine *Streptomyces* strain obtained from deep sea sediments, was used to isolate five structurally similar compounds which were found to have potent antifouling activity [84].

**Figure:**
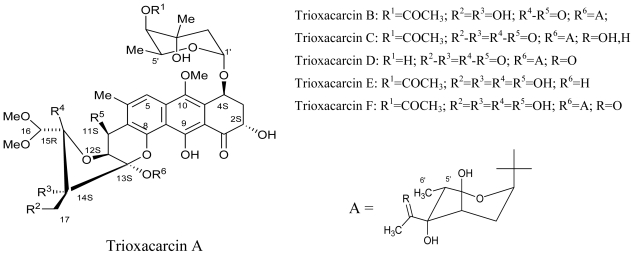


On the other hand, a marine-derived Actinomyces strain (NPS554) isolated from Japan yielded two trialkyl-substituted aromatic acids, Lorneic acid A and Lorneic acid B. It was observed that Lorneic acid A had significant inhibition activity against phosphodiesterase (PDE) 5 [85]. It is worth noting here that PDE5 inhibitors are of pharmacological importance in erectile dysfunctions and pulmonary hypertension.

**Figure:**
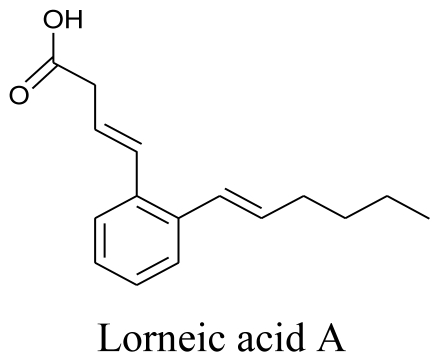


### 3.4. Microalgae

Cyanobacteria are a diverse group of Gram-negative bacteria, also known as blue-green algae that produce an array of secondary compounds with selective bioactivity against vertebrates, invertebrates, plants, microalgae, fungi, bacteria, viruses and cell lines [86]. Some reviews in the past have proved that they produce a wide variety of secondary metabolites with antifungal, antiviral, antibiotic and other activities, which make them an interesting candidate of potential pharmaceutical importance [87,88]. Certain anticancer compounds, which were initially thought to be obtained from marine sources, are now known to be produced by cyanobacteria [89]. Ulithiacyclamide and Patellamide A belong to Cyanobactins, produced by cyanobacteria, which have potent antimalarial, antitumor, and multidrug reversing activities [90].

**Figure:**
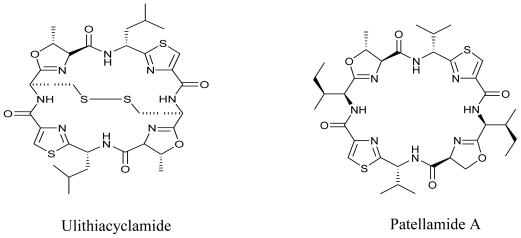


Recently, two new bioactive marine cyanobacterial natural products, Viridamides A and B, linear lipopeptides with a terminal acetylene group and novel terminal proline methyl ester and a 5-methoxydec-9-ynoic acid moiety, have been reported. It was observed that Viridamide A showed anti-trypanosomal activity with an IC_50_ of 1.1 μM and anti-leishmanial activity with an IC_50_ of 1.5 μM. The producer organism being blackish-green, mat-forming, filamentous cyanobacterium identified as *Oscillatoria nigro-viridis* [91]. Green algae have also been reported as producers of antiprotozoal molecules. In a recent study, Allmendinger *et al.* (2010) screened the antiprotozoal activity of crude extracts of four green marine algae (*Cladophora rupestris*, *Codium fragile sp. tomentosoides*, *Ulva intestinalis* and *Ulva lactuca*). All algal extracts showed antiprotozoal activity against *T. brucei rhodesiense*, and leishmanicidal activity with IC_50_ values ranging between 12.0 and 20.2 μg/mL [92]. This study was reportedly the first study to show antiprotozoal activity of British marine algae.

**Figure:**
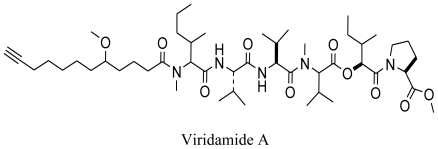


Diatoms are microscopic unicellular marine colonial algae belonging to the Diatomaceae family and having a siliceous very delicate covering [93]. In view of the growing concern for multidrug resistant bacterial strains, novel marine natural products from diatoms are being sought to combat this critical situation. Desbois *et al.* (2009) isolated an antibacterial polyunsaturated fatty acid, eicosapentaenoic acid (EPA) from the marine diatom, *Phaeodactylum tricornutum* Bohlin, which showed activity against a range of both Gram-positive and Gram-negative bacteria, including MRSA [94].

### 3.5. Marine viruses

Viruses are typically viewed as pathogens that cause disease in animals and plants. In recent years however, it has become increasingly clear that they play critical roles in the world’s oceans. Not much is known about the bioactive potential of marine viruses but the focus is turning these days to discover newer marine viruses and to study their physiological processes and the secondary metabolites produced, if any.

### 3.6. Symbiotic marine micro-organisms

Study of symbiotic microorganisms is a rapidly growing field as some reports from the recent past suspect that a number of metabolites obtained from algae and invertebrates may be produced by their associated microorganisms [35,95–98]. Sponge associated microbes are known for their tremendous activities covering a wide range of biological functions [99]. Recently, Baker *et al.* (2009) carried out a study aimed at isolation and identification of a diverse range of fungi from *H. simulans*. They used varieties of media for identification and determination of antimicrobial activities, if any. They isolated 19 different genotypes belonging to *Agaricomycotina*, *Mucoromycotina*, *Saccharomycotina*, and *Pezizomycotina;* some of these isolates showing antimicrobial inhibition of *Escherichia coli*, *Bacillus* sp., *Staphylococcus aureus*, and *Candida glabrata* [100]. The sponge-microbial association is a potential chemical and ecological phenomenon, which provides sustainable resource for developing novel pharmaceutical leads. Keeping in view the importance of antimicrobial potential of marine sponge associated microbes, targeting sponge microsymbionts is an essential focus nowadays [101,102].

A marine derived fungal strain M-3, isolated from marine red alga *Porphyra yezoensis* was screened for its antifungal activity (MIC-0.36 μM) against *Pyricularia oryzae* by Byun *et al.* (2003). As a result, a novel Diketopiperazine was isolated from the culture extracts and its structure was also elucidated by spectroscopic methods. A study in 2004, reported hemolysis and inhibition of *Candida albicans* by employing the butanolic extracts of the algal associated species *Pseudoalteromonas issachenkonii* cultures. Further analysis of the ethyl acetate extracts revealed that the basis of this antifungal activity was Isatin (indole-2,3-dione) [103].

**Figure:**
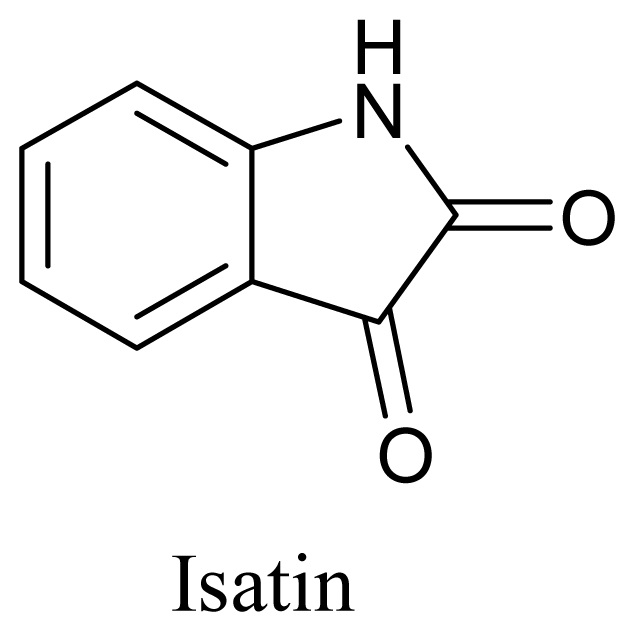


A red-brown hemolytic pigment of 269 Da and corresponding to molecular formula C_9_H_7_N_3_OS_3_ was also discovered by the same group [104]. Apart from great ecological value from the habitat point of view, coral reefs are the most species-rich environments in the oceans harboring a number of microbial species proving to be a rich and unexplored source of novel bioactive compounds [105].

Microbes associated with a number of marine organisms are also known for their bioactive potential [36]. About 10 years ago, Tapiolas and coworkers (1991) isolated and characterized two closely related novel compounds, Octalactins A and B from a marine derived *Streptomyces* sp. isolated from the surface of an unidentified gorgonian of the genus *Pacifigorgia*. They reported that Octalactin A exhibited strong cytotoxic activity towards B-16-FlO murine melanoma and HCT-116 human colon tumor cell lines with the IC_50_ values of 0.0072 and 0.5 pg/mL, respectively [106].

**Figure:**
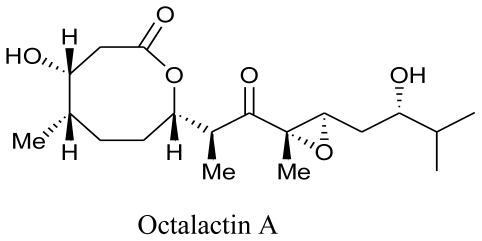


On similar lines, a recent study reported antibacterial and antilarval compounds from marine gorgonian associated bacterium *Bacillus amyloliquefaciens* SCSIO 00856 isolated from the South China Sea gorgonian, *Junceella juncea*. The broth of this strain showed strong antibacterial activity towards *Escherichia coli*, *Bacillus subtilis*, and *Staphyloccocus aureus* and antilarval activity towards the larvae of bryozoan *Bugula neritina*. When subjected to isolation procedures, a new 24-membered ring lactone, Macrolactin V was obtained from the culture broth [22].

Certain species of marine bacteria known as colonists of marine macro-organisms have also been reported to have antimicrobial potential. In a report, 42 strains of marine bacterial isolates showed antimicrobial activity and were assigned to genera *Alteromonas*, *Pseudomonas*, *Bacillus* and *Flavobacterium* [107]. Certain mycoparasitic and fungicolous fungi are known to colonize other fungal physiological structures and are known to be the source of effective bioactive agents [108–111]. A study conducted in the United States in 2002 reported five new natural products, Phomadecalins A, B, C and D, and Phomapentenone A, from cultures of *Phoma* sp. (NRRL 25697), a mitosporic fungal colonist isolated from the stromata of *Hypoxylon* sp. These compounds were characterized structurally and four compounds (phomadecalins A–D) were found to be active against Gram-positive bacteria, *Bacillus subtilis* (ATCC 6051) and *Staphylococcus aureus* (ATCC 29213) [112].

**Figure:**
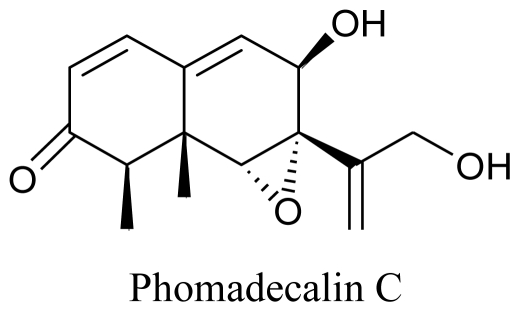


The saga of marine microbial bioactive metabolites is continuing with new compounds being added day by day. Our knowledge, however, is still a miniscule of what exists deep in the oceans. Since it is almost impossible to cover all the marine derived microbial bioactive substances in a single review, the representative chemical structures of some of the above mentioned secondary metabolites/bioactive substances have been presented.

## 4. Clinical Status of Marine Compounds of Microbial Origin

The ultimate goal of the bioactivity driven drug discovery from marine sources is to discover newer and effective formulations for the treatment of deadly human diseases. The first defined marine product to enter clinical trials for any major human disease was Didemnin B isolated from the tunicate *Trididemnum solidum*. Since then, a number of drugs such as Ara A (Vidarabine-Vira-A^®^), Ara C (antiviral) and synthetic analogue of Ara C- cytarabine (Cytosar-U^®^-anticancer) have successfully passed the trials to become clinically approved drugs of pharmaceutical importance [113]. Apart from this, Yondelis^®^, otherwise known as Ecteinascidin 743, has been approved in Europe for the treatment of soft tissue sarcoma and another marine bioactive metabolite, the Conus toxin, also known as Ziconotide or Prialt^®^ has been approved safe for intractable pain [114].

Despite the vast diversity of microbial consortium in deep oceans and the plethora of bioactivity that they display, very few drugs of marine microbial origin are on the market. With an integrated approach of microbiology, screening and natural products chemistry, the marine microbial metabolites are now advancing to be pharmaceutically important drug candidates. Plinabulin (NPI-2358), a potent and selective vascular disrupting agent (VDA) initially isolated from a marine fungal extract is presently undergoing Phase II clinical trials. Preclinical studies suggested this compound to be active against multi-drug resistant human tumor cell lines and it was observed to greatly enhance the efficacy when used in combination with the already existing chemo- or radio-therapy regimens in a variety of animal models [114,115]. Tasidotin, Synthadotin (ILX-651) derived from a marine bacterium is also under Phase II clinical trials with Genzyme Corporation (Cambridge, MA). Another bacterial peptide of marine origin, Soblidotin (TZT 1027), is undergoing Phase III clinical trials under the supervision of Aska Pharmaceuticals (Tokyo, Japan). Both of these compounds are promising anticancer agents.

**Figure:**
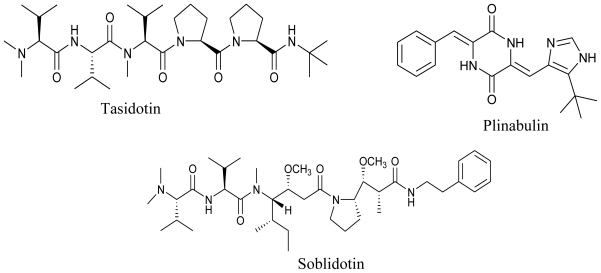


One of the most sought after marine microbial drug candidates is Marizomib (Salinosporamide A; NPI-0052). Salinosporamide A is a novel proteasome inhibitor isolated from a marine bacterium *Salinispora tropica* [116] and is undergoing Phase I clinical trials under the aegis of Nereus Pharmaceuticals (San Diego, CA). Interesting properties such as a broader and longer lasting proteasome inhibition, efficacy against a wider range of hematologic malignancies and many solid tumor models, less cytotoxicity to normal cells, higher *in vivo* potency and potential for both oral and intravenous administration makes Salinosporamide A a very promising anticancer agent [115]. Bryostatin 1 is a well known marine anticancer agent, initially thought to be produced by Bryozoa. However recent reports have confirmed its production from the bacterial symbiont, *Candidatus Endobugula sertula* [117]. This compound is also under Phase I clinical trials with National Cancer Institute (NIH, U.S.) [114]. Sorbicillactone-A is yet another antileukemic agent produced by *Penicillium chrysogenum* associated with marine sponge *Ircinia fasciculata.* This alkaloid is also known to have antiviral and neuroprotective properties [118]. Owing to its wonderful antileukemic properties, this compound has also qualified for human trials [119].

**Figure:**
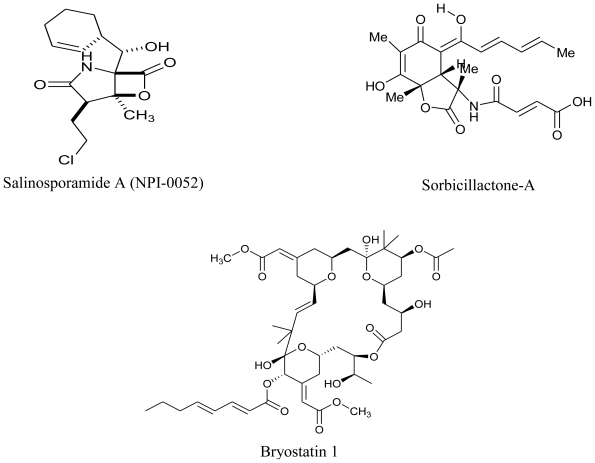


Cyanobacteria produce a family of antitumor agents known as Cryptophycins, which interfere with the tubulin assembly. A synthetic cryptophycin derivative (LY355703, CRYPTO 52) is in the early stages of clinical evaluation. Depsipeptide (NSC 630176) is a bicyclic peptide isolated from *Chromobacterium violaceum.* It decreases mRNA expression of the *c-MYC* oncogene, causes cell-cycle arrest at G0-G1 and acts as an inhibitor of a histone deacetylase. These properties make it a promising anticancer agent for which the Phase I clinical trials are soon to begin [120]. With augmenting focus of research in the field of marine biotechnology, novel and potent compounds of biomedical interest are being discovered from the ocean bed, at a speedy rate. The most efficient and bioactive substances would keep on qualifying for clinical trials to become successful drug candidates in future.

## 5. Future Perspectives and Concluding Remarks

As evidenced from past and ongoing research, microbial consortium has an excellent plethora of bioactivity. A number of past reviews have focused the attention of researchers on this tremendous treasure of the marine microbial environment [25,35,36,95–98]. But there is still a long way to go. Although a diversified range *viz.*, antibiotic, antifungal, cytotoxic, neurotoxic, antimitotic, antiviral, antineoplastic and antiprotozoal activity is known, extensive studies are still needed in the field of AIDS, immunosuppression, anti-inflammation, Alzheimer disease and ageing processes [121]. Efforts are needed to expand the marine microbes derived drug discovery to include other diseases, which need extensive and urgent attention in terms of new therapies. The approach needs to be more focused and organized to combat multi drug resistance and a serious threat of re-emerging infectious diseases, which is a growing concern in the medical fraternity.

Interdisciplinary research and collaborative endeavors are required amongst scientists, medical practitioners, marine microbiologists and biotechnologists, to provide innovative approaches to marine based biomedical research. As discussed in the introduction section, the marine organisms are already being used as biomedical models to understand the complexity of disease processes in humans [5], but the role of marine microbes in the same field has not been well explored. Knowledge about the exact mechanism of action of the microbial metabolites, cross talk between marine sciences and microbial biotechnology, proper understanding of the chemical interactions in the oceanic environment and development of appropriate bioassays may be utilized for development of newer classes of marine microbes derived drugs.

Advances have begun in the field of marine microbial biotechnology but a more extensive and focused approach is needed to investigate what else the marine microbes have to offer. A recent report suggested the production of bioactive peptides from marine yeast *Aureobasidium pullulans* HN2-3. The peptides produced from single-cell protein of marine yeast strain G7a had good angiotensinconverting enzyme inhibitory activity [122]. Thorough research in the field of bioactive peptides from marine microbes may open the gates for many future implications in the field of biomedical sciences. In fact, microbial biofilms also have a number of technological applications which could be looked into. Many signal transduction pathways involved in the production of marine microbial antifouling compounds can be worked out through a more specific research on biofilms. It is believed that once genetic regulation of the colonization process is better understood, chemicals can be identified and applied that will directly interfere with genetic transcription itself or inhibit or foster any step along the signal transduction pathway [123].

As any other chemical reaction or metabolic process, production of secondary metabolites also depends upon certain physico-chemical factors *viz*., amount of oxygen available, optimum temperature and pH. The appropriate maintenance of these parameters may enhance metabolite production. Moreover, the pharmaceutical industry is now concentrating on traditional mutagenesis programs for strain and product yield improvement. Genetic engineering could prove to be a boon for improvement in the yield of bioactive metabolites as the biosynthetic pathways can now be manipulated through recombinant DNA technology. In addition, researchers are now trying for heterologous expression of biosynthetic gene clusters in other organisms, which would not only increase the production levels but also speed the process by using rapidly growing and easy to manipulate organisms compared to the producing organism [124].

It is worth giving serious consideration to the exploitation of marine microbial life and the associated secondary metabolites, aided by genomic analyses, applying metabolic approach and employing combined biomedical and biotechnological efforts, which would lead to discovery of some novel, lead compounds of a varied degree of bioactivity. The novel bioactive metabolites isolated and characterized from marine microbes would be useful in controlling human diseases and protecting human health by solving tribulations associated with antibiotic resistance. Certain bioactive metabolites may also be beneficial in ensuring environmental hygiene (antifouling compounds). The development of more automated and more affordable techniques for isolating and characterizing marine microbial bioactive metabolites would definitely make marine microbial natural product extracts more accessible to natural products’ chemists and make life more disease free and worth living for mankind.

## Figures and Tables

**Table 1:** Structure and biological activity of some of the marine microbial metabolites.

Microorganism	Compound	Biological activity	Reference
***Cyanobacteria***			
Dolastatin 10	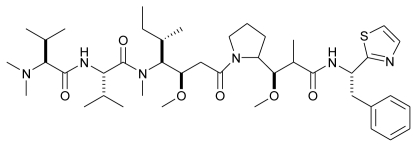	Antimicrotubule; and the synthetic analogue, TZT-1027, as antitumor	
Dolastatin 15	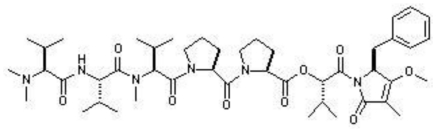	Antimicrotubule; and the synthetic analogue, ILX-651, as antitumor	[7]
Curacin A	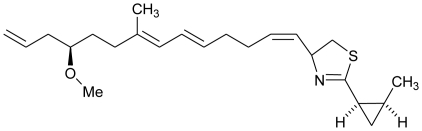	Antimicrotubule	
Toyocamycin	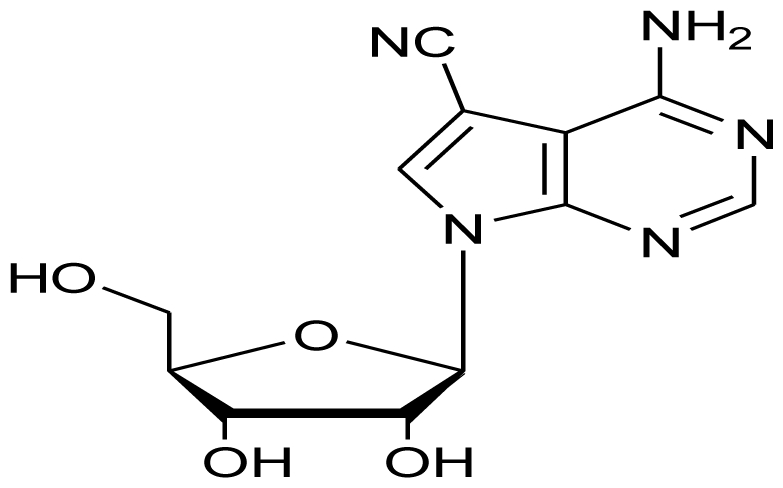	Antifungal	[8]

***Actinomycetes***			
Resistoflavine	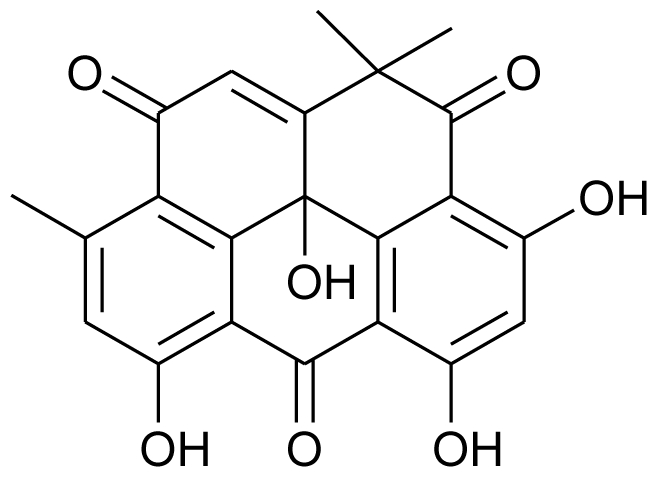	Anticancerous and antibacterial	[9]
Marinomycin A	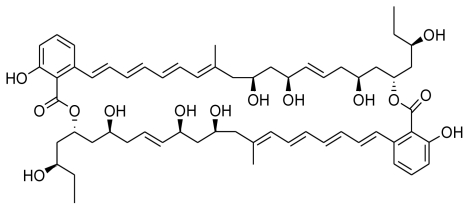	Antitumor and antibiotic	[10]
Daryamide C	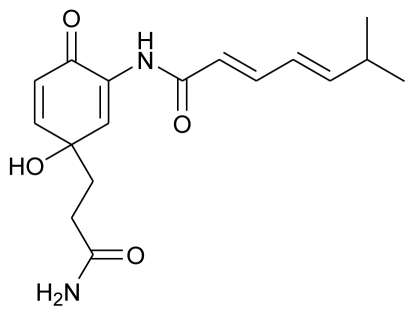	Antitumor	[11]
Violacein	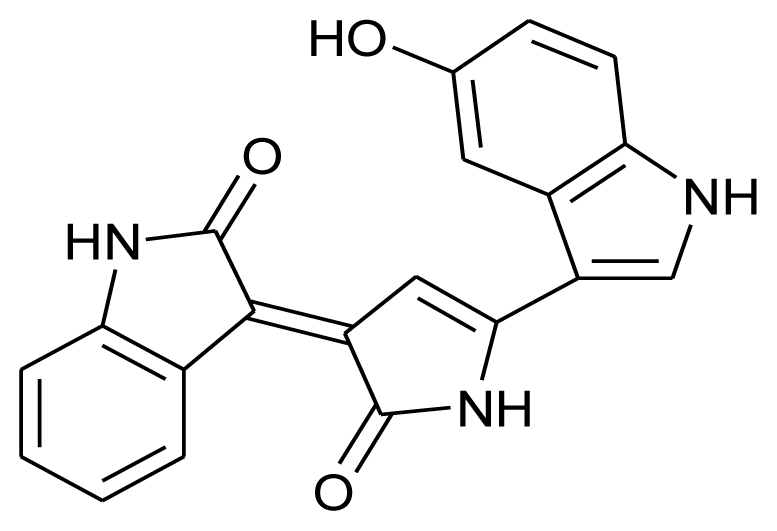	Antiprotozoal	[12]

***Bacteria***			
Macrolactin S	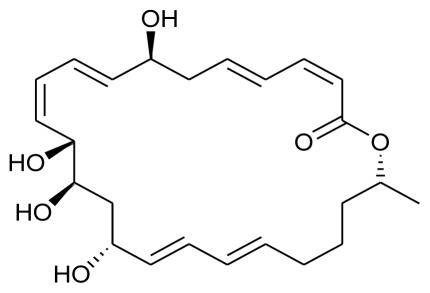	Antibacterial	[13]
Pyrone I and II	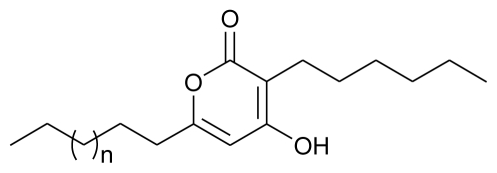	Antibacterial	[14]
MC21-B	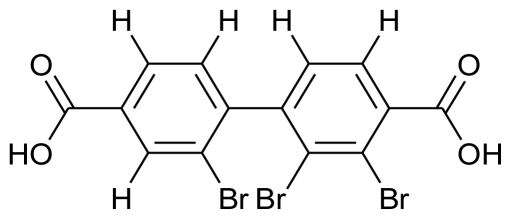	Antibacterial	[15]

***Fungi***			
Meleagrin	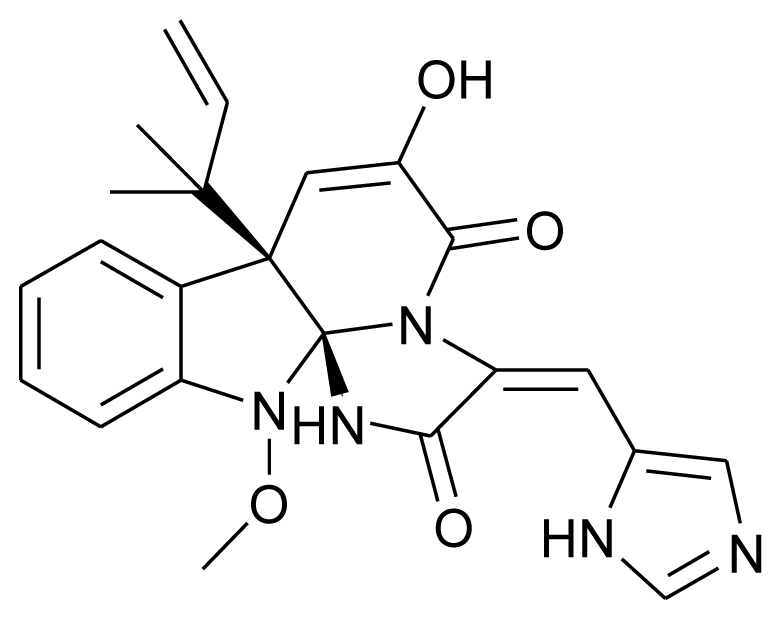	Antitumor	[16]
Oxaline	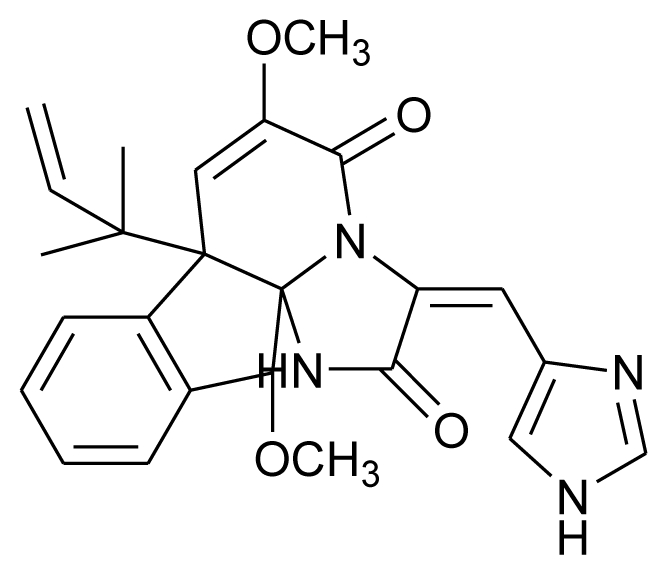	Antitumor	[17]
Alternaramide	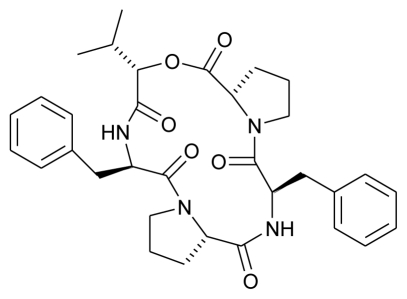	Antibacterial	[18]

***Algae***			
Norharman	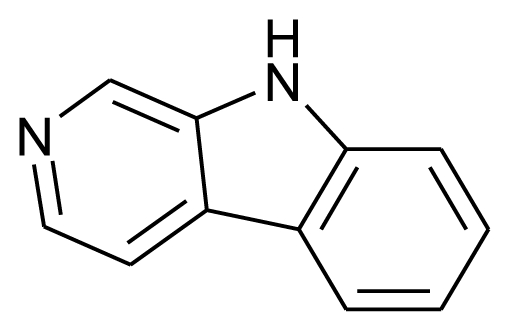	Enzyme inhibitor	[19]
Calothrixin-A	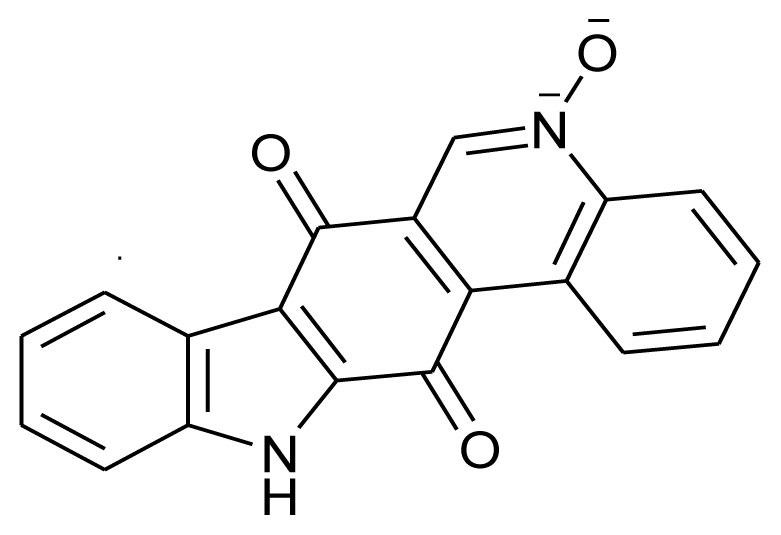	Antimalarial and anticancerous	[20]
Eicosapentanoic acid (EPA)	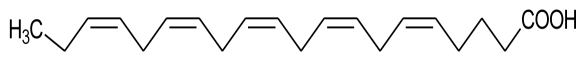	Treats heart disease, Anti inflammatory agent (rheumatoid arthritis and immunodeficiency diseases)	[21]

***Symbiotic microbes***			
Macrolactin V	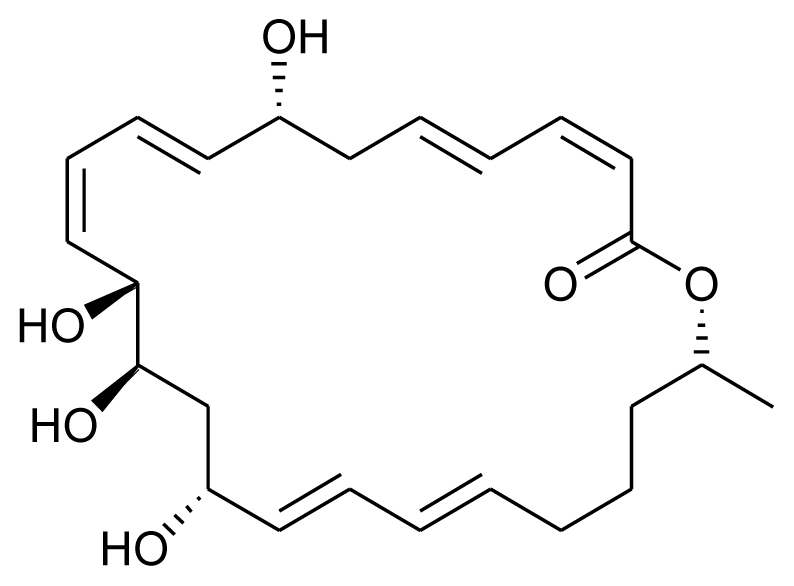	Antibacterial and antilarval	[22]
DAPG	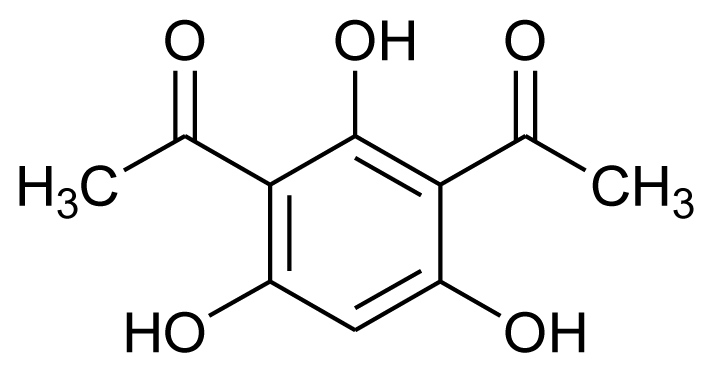	Antibacterial (anti-MRSA, anti-VRSA and anti-VRE)	[23]
BE-43472B	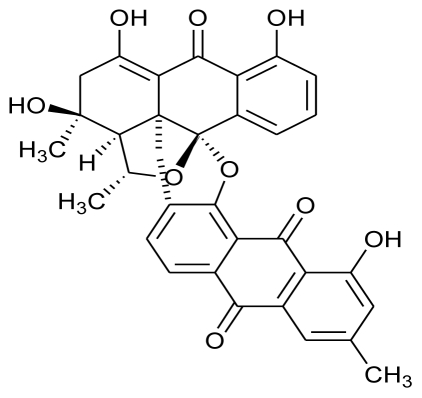	Antibacterial (anti-MRSA and anti-VRE)	[24]
